# Same ages, different stages: Pubertal development in 9- and 10-year olds is associated with entropy of fMRI signals

**DOI:** 10.1016/j.dcn.2026.101806

**Published:** 2026-09-01

**Authors:** Kirsten M.P. McKone, Donovan J. Roediger, Stefanie L. Sequeira, Ellery Island, Monica Luciana, Mark B. Fiecas, Bryon A. Mueller, Bonnie Klimes-Dougan, Kathryn R. Cullen

**Affiliations:** a University of Minnesota Institute of Child Development, USA; b University of Minnesota Department of Psychiatry & Behavioral Sciences, USA; c University of Virginia Department of Psychology, USA; d University of Minnesota Division of Biostatistics, USA; e University of Minnesota Department of Psychology, USA

**Keywords:** Entropy, Pubertal development, Adolescence, ABCD Study, Brain development, Complexity, RsFMRI BOLD signal

## Abstract

Entropy is a novel brain measure that holds important information about brain flexibility and functioning, with promise for informing brain development in adolescence, as it has been shown to change across the lifespan. However, little is known about how it changes during the critically important phase of pubertal development, which kickstarts a period of broad neural reorganization and restructuring. This proof-of-concept cross-sectional study examined associations between fMRI signal entropy and early pubertal development in a large, diverse sample of children. Data (*N* = 6838; 49.6% male; ages 8.92–11.08) was drawn from the first wave of the Adolescent Brain Cognitive Development study. Sample entropy was calculated on the time series of the resting-state fMRI signals for every grayordinate (i.e., cortical vertex and subcortical voxel). Parent-reported pubertal development (approximated Tanner stage) and salivary hormone levels (dehydroepiandrosterone; estradiol; testosterone) were correlated with sample entropy across the whole brain. The patterns of association between sample entropy and pubertal stage/hormone levels were inspected for males and females separately. Findings indicated pubertal development was associated with higher levels of resting state fMRI signal entropy, specifically in the occipital, parietal, and temporal lobes. Associations were apparent in females across most pubertal metrics. For males the constrained range of pubertal development may in part account for weaker associations observed. Further, adjustment for variables associated with early timing of pubertal development (socioeconomic status, body mass index) reduced the strength of puberty-entropy associations for both sexes. Longitudinal research is needed to confirm the associations between sample entropy and puberty, to evaluate whether stronger associations emerge as more males enter puberty in earnest, and to disentangle potential confounds.

## Highlights

• This study correlated brain entropy and pubertal development in children ages 9–10.
• Entropy increases with both self-reported and hormonal measures of puberty.
• Effects are strongest in brain areas associated with sensory and social processing.
• Females had greater variability in pubertal development and showed stronger effects.

## Graphical Abstract

**Figure:**
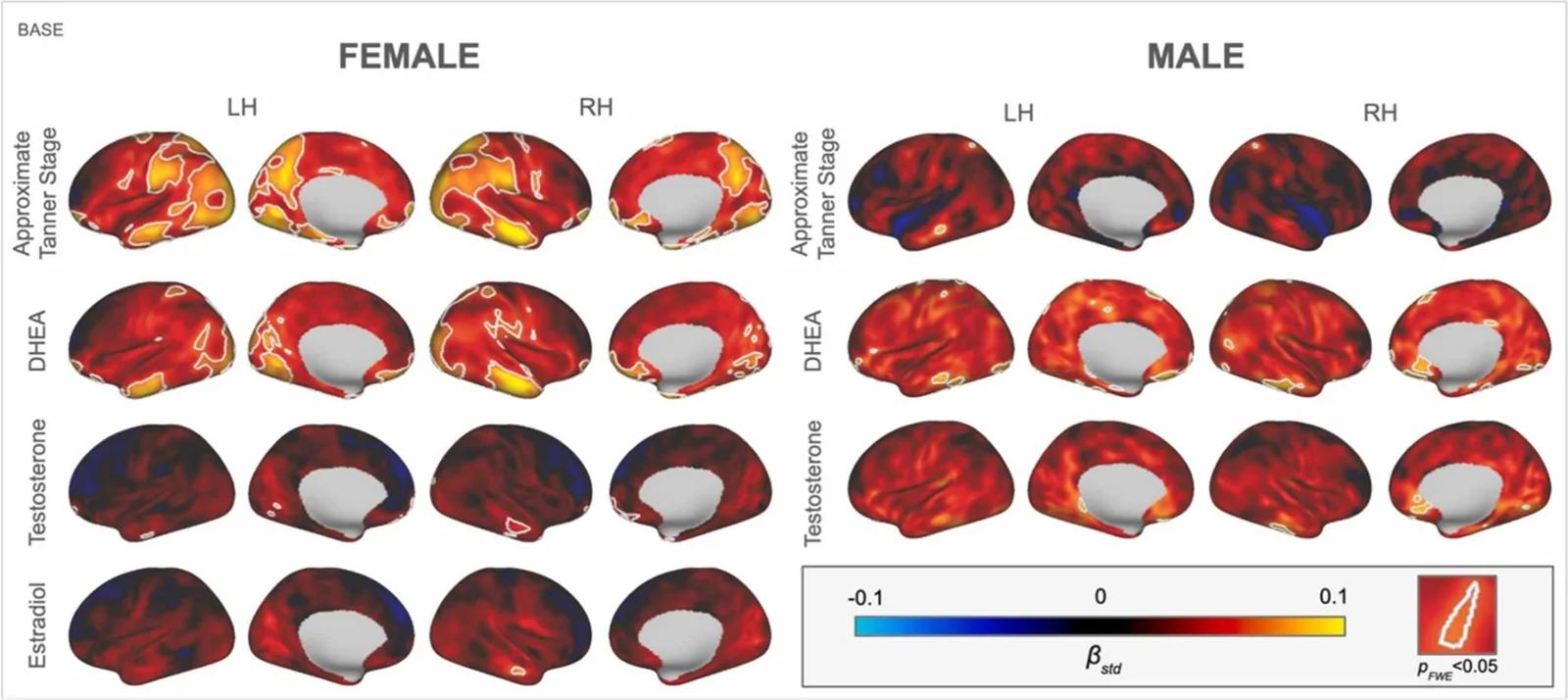
Entropy of the fMRI Bold signal is positively associated with pubertal development in adolescents ages 8.92–11.08, with the strongest associations in females, who are more advanced in pubertal development at this age, and in areas associated with sensory and social processing (occipital, parietal, temporal lobes). *(Note.* DHEA = Dehydroepiandrosterone.)

## 1 Introduction

Adolescence is a period of intensive growth and change in brain structure and function, changes associated with increases in social, emotional, and reward sensitivity in adolescents (e.g., Blakemore and Mills, 2014; Crone and Dahl, 2012; Forbes andDahl, 2010; Nelson et al., 2005). Scholars have proposed that these brain changes make adolescence a time of greater cognitive and behavioral flexibility (Crone and Dahl, 2012, Larsen and Luna, 2018, Piekarski et al., 2017). Such theories posit a core role for puberty in “opening” neural systems within the adolescent brain and increasing its context-sensitivity, priming it for substantial experience-dependent learning (Luciana and Collins, 2021). From this vantage, puberty-spurred adolescent brain development lays the groundwork for effective learning from the environment as adolescents transition from their families of origin and are asked to adapt to the novel relationships, roles, and independence of adulthood. Despite increasing recognition of the dynamic nature and complexity of adolescent behavior, there has been little research into dynamic measures of brain function that may underpin these observed developmental changes in adolescence. Recent research illustrates that the dynamic complexity of brain signals holds important information about brain function (e.g., Carhart-Harris et al., 2014; Cieri et al., 2021; D.J.J. Wang et al., 2018), and may provide insight into adolescent brain development. In the current study, we evaluate how one measure of dynamic brain signal complexity, entropy of the fMRI brain oxygen level dependent (BOLD) signal, relates to early pubertal development.

### 1.1 Entropy of fMRI BOLD signals: a measure of complexity

Researchers have theorized that brain entropy may relate to criticality (e.g., Carhart-Harris and Friston, 2019; D. J. J. Wang et al., 2018) – or the brain existing in a condition at the interstice of order and chaos – a steady state that facilitates changes in the configuration of brain activity. Entropy, including that of the fMRI BOLD signal, has theoretical grounding in both information theory and biological dynamic systems theory. From the perspective of information theory, entropy is operationalized as the unpredictability of a signal over time, with more unpredictable signals carrying more information (Keshmiri, 2020). Dynamic systems theories, by comparison, posit that systems with higher entropy are more complex or more irregular, and are therefore more adaptable to changes in the environment compared to more rigid systems (Carhart-Harris and Friston, 2019); from this perspective, more entropic brain signals are more flexible in response to environmental inputs. A burgeoning literature has emerged leveraging fMRI signal entropy derived in a variety of experimental settings, including during the resting state and functional tasks (e.g., Amalric and Cantlon, 2023; Niu et al., 2022; D. J. J. Wang et al., 2018; N. Wang et al., 2018). There are a number of methods leveraged to calculate fMRI signal entropy (e.g., sample entropy, multiscale entropy, permutation entropy; Cieri et al., 2021), but they all broadly seek to capture the amount of complexity in the fMRI signal. In the case of sample entropy, a pattern-matching algorithm is applied to the timeseries of the fMRI BOLD signal, with less patterned (i.e., less repetitive, more stochastic) signals considered more highly entropic and complex (see Roediger et al., 2024 for illustration).

Emerging research indicates that entropy is related to both state- (Cieri et al., 2021) and trait-level characteristics (DeYoung and Krueger, 2018), as well as recovery processes (Carhart-Harris and Friston, 2019). At the state level, studies have illustrated that waking states exhibit higher entropy than deep sleep states (Cieri et al., 2021), and states induced by psychedelic drug use exhibit higher entropy than typical brain states, with the strength of the effect associated with the subjective intensity of the drug experience (Carhart-Harris et al., 2014). At the trait level, higher brain entropy has been linked to higher levels of intelligence (Saxe et al., 2018), divergent thinking and creativity (Shi et al., 2019), and bilingualism (Grundy et al., 2017). Scholars also highlight the potential role for brain entropy in supporting neural plasticity and restructuring (Carhart-Harris and Friston, 2019).

### 1.2 Entropy and lifespan brain development

To date, few studies have examined fMRI signal entropy in association with development in childhood and adolescence. However, a few have examined measures of fMRI-based entropy in the perinatal period, with a view of elucidating the potential role of entropy in the developing brain, which has been theorized since the mid-20th century (Bergström, 1969, Bergström, 1974). Considering the perinatal period and adolescence both feature rapid brain development with specificity based on biological sex, insights on entropy from the perinatal period may inform conceptualizations of entropy and pubertal development in adolescence. Zhao and colleagues (2024) conducted a developmentally sensitive examination of entropy in the perinatal brain, finding entropy was differentially associated with overall age, gestational age, and post-natal age, that taken together suggest entropy is influenced by both biology and experience. Most relevant to the current study, whereas entropy in the sensorimotor-auditory and association cortices was *negatively* correlated with gestational age, entropy in these regions was *positively* correlated with postnatal age. Additionally, pre-term infants showed higher entropy values, especially in the visuomotor cortex, compared to infants who were born at term. The researchers interpreted these distinct age associations as indicating that some facets of entropy were biologically based (i.e., genetic) and some experience-dependent (e.g., post-natal age associations), perhaps due to myelination and increased connectivity. These findings mirror the spatiotemporal development of the infant brain, and were theorized by the authors to represent “heightened neural flexibility to adapt to ex-utero environment” (p. 8; Zhao et al., 2024). Entropy also appears to increase alongside functional development in childhood: Amalric and Cantlon (2023) found that children ages 9–10 who exhibited performance similar to adults on specific learning tasks also exhibited similarly adult-like entropy levels in corresponding functional areas of the brain (Amalric and Cantlon, 2023), suggesting that entropy may increase alongside functional ability in childhood, possibly due to processes related to connectivity, synaptogenesis, and/or myelination during the learning process.

In addition to this nascent research examining fMRI BOLD signal entropy alongside infant and child development, some research has attempted to characterize developmental changes in brain signal entropy across the lifespan. Many such studies have focused exclusively on adults (Cieri et al., 2021); however, several have examined entropy and age associations cross-sectionally from childhood through adulthood. These studies indicate entropy exhibits relatively sharp increases throughout early life, until approximately midlife (i.e., ages 30–50), after which it shifts to a shallow decline through the end of life (Fan et al., 2017, Niu et al., 2022, Yao et al., 2013). Findings on decreasing entropy during normative and pathological aging processes have led some to theorize that such decreases in entropy are related to decreasing sensory capacity in the aging brain (Cieri et al., 2021). Despite these initial indications of age-related increases in entropy from childhood through early adulthood each study operationalized entropy and modeled age-related change in a different way, inviting more research to further clarify the developmental picture of entropy of fMRI signals. Further, likely due to limitations in sample size, most studies aggregated all participants in childhood and adolescence with young adults, limiting their ability to sensitively evaluate developmental differences in entropy throughout childhood and adolescence. However, as discussed below, it may be particularly informative to examine developmental changes in entropy across the period of rapid brain development associated with pubertal development.

### 1.3 Puberty kickstarts rapid brain development

The physical and hormonal changes of puberty are theorized to initiate a cascade of neural, social, and behavioral changes during the adolescent period. Several theories of adolescent brain development highlight puberty as a second period of rapid sex-based brain development (after the perinatal period; Schulz et al., 2009). Further, some scholars theorize that the wide-ranging nature of puberty-spurred changes in the adolescent brain result in adolescence being a developmental stage marked by sensitivity to context (Crone and Dahl, 2012). The convergence of these theories aligns with notions of brain entropy in adolescence potentially playing a role in experience-dependent development, parallel to that observed in the perinatal brain (Amalric and Cantlon, 2023, Zhao et al., 2024). Further, evidence from the developmental entropy literature suggests that entropy is intimately tied to sensory integration both in infancy and aging (Cieri et al., 2021, Zhao et al., 2024). Considering theories of adolescence as a time of increased sensitivity to environmental input spurred by hormonal development at puberty (Crone and Dahl, 2012) and research indicating considerable development along the sensorimotor-association axis during puberty (e.g., Larsen et al., 2023), it is plausible that puberty may spur considerable change in brain entropy as well.

Puberty occurs through two distinct but overlapping processes. The first process, adrenarche, onsets in mid- to late-childhood, and is predominantly associated with an increase in peripherally circulating and brain-synthesized dehydroepiandrosterone (DHEA), which plays a role most notably in increases in body odor, body hair growth, and growth in height (Byrne et al., 2017). The second pubertal phase, gonadarche, begins approximately two years earlier in girls than boys (e.g., Berenbaum et al., 2015) and is associated with the emergence of secondary sex characteristics. Considerable research on puberty’s impact on brain development has focused on gonadarche, under the assumption that the surge of sex hormones, testosterone and estradiol, would spur much of the structural and functional brain alterations observed at that time (e.g., Beltz et al., 2013; Beltz and Berenbaum, 2013; Schulz and Sisk, 2016). As a result, such research has historically examined pubertal influences at later stages of pubertal development (i.e., exclusively during gonadarche/the teenage years). However, more recently, some have raised the possibility of DHEA and processes related to adrenarche performing an important role in brain development (Barendse et al., 2018, Byrne et al., 2017). As the extended adrenarche experienced by humans is shared almost exclusively by higher-order primates (Byrne et al., 2017, Conley et al., 2012), some theorize that adrenarche/DHEA may serve a critical role in supporting higher-order cognition and lay the foundation for the sensitive period of brain development observed in adolescence, through increases in plasticity mediated by DHEA (Byrne et al., 2017, Cumberland et al., 2021). Hence, there is a need for research on puberty and brain development that is focused on the earliest stages of puberty, when there may be increases in circulating and brain-synthesized DHEA due to adrenarche, but youth may not yet be exhibiting the externally observable physical changes of puberty associated with gonadarche.

Although youth tend to progress through the various stages of puberty in similar order, there are considerable individual differences in the timing of pubertal onset (i.e., pubertal timing, beyond sex differences in timing of onset) and how quickly (or slowly) they progress through the various stages of puberty (i.e., pubertal tempo). Hence, it is important to examine group-level associations between puberty and brain development in sufficiently large samples, to account for the variability both within and across adolescents. Further, recent research suggests multiple indicators of pubertal development provide unique information about the role of puberty in brain development, including pubertal status (i.e., an individual’s overall pubertal development, typically tracked through observable features such as body hair growth, height, and, for females, menstruation) and the influence of specific pubertal hormones, including DHEA, testosterone, and estradiol. Multimethod measurement of puberty may be particularly valuable when studying brain development in association with the earliest stages of pubertal development, since, as noted above, there may be brain changes associated with alterations in DHEA prior to the rise of estradiol and testosterone, or before the influence of hormones is visible in physical indicators used to evaluate pubertal status.

Conceptualizing adolescence as a time of increased sensitivity to context and subsequent flexibility and adaptability, spurred by the biological changes of puberty, points to the potential for changes in entropy during this key developmental transition. Hence, it is important to understand associations between early pubertal development and entropy, considering signal complexity may play a prominent role in brain development and/or serve as a valuable measure to enhance our understanding of brain development. Some have theorized a role of entropy in the development of cognitive flexibility throughout adolescence (Parr et al., 2024), highlighting the need to understand the role of entropy through pubertal development to provide the construct with a firm developmental grounding. However, to date no studies have examined associations between pubertal development and entropy. Further, limitations in measuring puberty in a sensitive manner have stifled progress in understanding puberty’s effects on the brain, leading to calls for more a sensitive approach to puberty and brain development (Cheng et al., 2020, Vijayakumar et al., 2018). Gaining a nuanced understanding of associations between pubertal development and entropy will likely require a multimethod approach to the assessment of puberty, including both more fine-grained evaluation of pubertal hormones, as well as examining associations with broader measures of pubertal development, such as pubertal status (Cheng et al., 2020).

### 1.4 The current study

The current study sought to evaluate whether there is an association between entropy of the fMRI BOLD signal during the resting state and stages of early pubertal development during late childhood in the Adolescent Brain Cognitive Development (ABCD) Study® sample, a large, diverse sample designed to examine longitudinal brain and cognitive development. We adopted an exploratory, descriptive approach, examining entropy across the whole brain and in association with two primary indicators of pubertal development, pubertal status and hormone concentrations (Cheng et al., 2020), and considering distinctions between age and pubertal development. To this end, we examined associations separately by sex, not because we expected to see sex differences in brain entropy, per se, but because there may be differences in brain entropy based on sex due to developmental differences in the timing of puberty. We opted to use sample entropy (SampEn) as our operationalization of entropy given its wide usage, its simplicity, replicability and interpretability (Roediger et al., 2024).

## 2 Method

### 2.1 ABCD study procedure

This study leveraged data from the first wave (Baseline) of the ABCD Study, which was conducted between September 2016 and October 2018 (Garavan et al., 2018). All data were drawn from the 5.1 release. Demographic and developmental data were drawn from the primary release; resting state fMRI data were used to generate entropy estimates following a modified preprocessing pipeline as detailed below. The overarching sample comprised *N* = 11,880 children ages 9–10 recruited by 21 sites across the contiguous United States. Research ethics approval was conducted through a centralized IRB process at the University of California, San Diego, with individual study sites obtaining local IRB approval in addition. Caregivers provided written informed consent and each child provided written assent. Of particular relevance to the current study, as part of the standard procedures of the ABCD Study, children and their participating parent(s) completed a series of self-report questionnaires, including on pubertal development. Children also completed a saliva collection, from which hormones were assayed, and resting state fMRI scans (see below for detail).

### 2.2 Design considerations and sample size

**Fig. 1:**
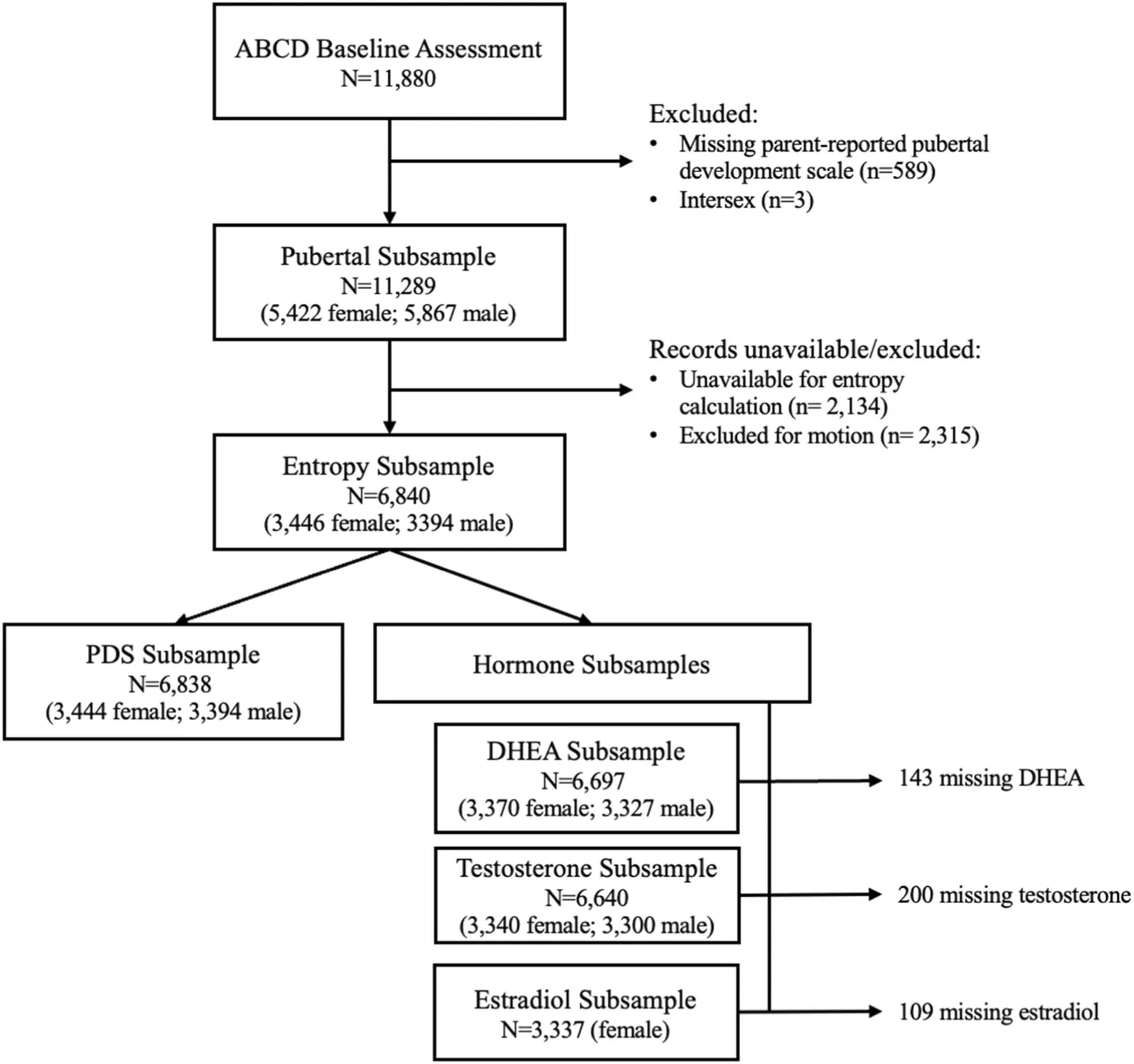
Flow Diagram of Participant Missingness.

In the current study, we included all participants who had valid entropy and parent-reported pubertal status data. A flow diagram specifying participant exclusions is presented in Fig. 1. As we intended to evaluate sex-based hormones and pubertal development, three participants who self-reported as intersex were excluded. The final analysis sample comprised *N* = 6838 youth ages 8.92–11.08, 49.6% of whom were assigned male at birth.

### 2.3 Measures

#### 2.3.1 Sample entropy

Sample entropy was calculated from the resting-state fMRI. Resting-state fMRI acquisitions in ABCD typically consisted of four 5-minute runs, during which participants were instructed to view a fixation cross. Imaging data were preprocessed by the ABCD Consortium using the ABCD-HCP pipelines (Feczko et al., 2021, Hagler et al., 2019), with modifications to the DCANBOLDproc stage to include a wider bandpass filter (0.009–0.25 Hz) and additional spatial smoothing (4 mm Gaussian, applied separately in surface and volume spaces) to preserve higher frequency signal dynamics.

Sample entropy of the BOLD signal was calculated at every grayordinate (i.e., cortical vertex and subcortical voxel) using the powseR package in R (Roediger et al., 2024). The powseR package was developed to facilitate the calculation of SampEn in contemporary neuroimaging data, address motion, and identify optimal parameters. To mitigate motion artifacts, which can artificially depress sample entropy (De Vries et al., 2020), powseR identified and extracted 20 low motion windows from across all available (typically four) 5-minute runs. The program was designed to identify the most usable (i.e., low motion) data while preserving continuity of the windows selected. Each of the 20 selected windows consisted of 20 uninterrupted volumes with respiratory-filtered framewise displacement (FD) below 0.2 mm for a total of 400 volumes per participant, with censored timepoints inserted at the window junctions as buffers to prevent artificial temporal continuity. Entropy calculations explicitly ignored these inter-window buffers following the method described in (Dong et al., 2019). As a result, all entropy calculations were performed on time series of matching record length and contiguity. From these extracted windows, powseR computed SampEn utilizing data-driven optimal parameters for template length (*m*=2) and tolerance factor (*r* = 0.325; see Roediger et al., 2024 for detail on how the powseR package identifies optimal parameters from the data), which were then leveraged in the calculation of entropy, as described subsequently.

Sample entropy was calculated using the calc_entropy function in powseR, which is based on Richman and Moorman’s method to resolve issues inherent to approximate entropy, specifically its self-matching bias and dependence on time-series length (Richman and Moorman, 2000). Mechanically, SampEn evaluates all possible signal templates of length *m* and identifies matching templates across the time series within a defined tolerance, *r*, explicitly excluding self-matches. It then determines what proportion of these *m*-length matches continue to match when the template length is extended to *m+*1. The mathematical operationalization of Sample Entropy is the negative natural logarithm of this conditional probability. In descriptive terms, if a high proportion of matching templates at length *m* also match at length *m*+1, the signal is more predictable and therefore less complex (Roediger et al., 2024). We further relied on Connectome Workbench (Marcus et al., 2011) to visualize the resultant estimates.

#### 2.3.2 Pubertal status (Approximated Tanner Stage)

As part of the baseline ABCD Study procedures, participants and their parents completed the Pubertal Development Scale (PDS; Petersen et al., 1988), which assesses a range of pubertal development indicators, some that apply to both sexes (i.e., growth spurt, skin changes, body hair growth) and some that are specific to females (i.e., breast development) and males (i.e., deepening voice, facial hair growth). Items were rated on a 1–4 scale where 1 = process has not yet begun, 2 = process has barely started, 3 = changes are definitely underway, and 4 = process seems complete. For females, menarche was scored separately in a dichotomous fashion (1 = no; 4 = yes). For the current study, we leveraged the pubertal category score measure approximating the child’s Tanner stage, which was provided in the tabulated data and derived from the PDS (Herting et al., 2021). For females, the items assessing body hair growth, breast development, and menarche were summed; for males, the items for body hair growth, voice change, and facial hair items were summed. Final pubertal category scores were as follows: 1 = prepubertal; 2 = early pubertal; 3 = midpubertal; 4 = late pubertal; 5 = postpubertal. Caregiver reports were prioritized at the baseline timepoint used here, as child participants provided a high degree of “I don’t know” responses. Pubertal status was modeled as a continuous variable.

#### 2.3.3 Salivary Pubertal Hormones (DHEA, Testosterone, Estradiol)

As part of standard ABCD Study procedures, saliva was collected via passive drool with assistance from trained research assistants, (see Cheng et al., 2020, Herting et al., 2021), and three pubertal hormones were assayed in duplicate: DHEA and testosterone in males and females, and estradiol in females only. The decision tree for establishing a single value and to exclude outliers outlined in (Herting et al., 2021) was followed using the script referenced in Barendse and colleagues (Barendse et al., 2024).

#### 2.3.4 Hormone covariates

We also included several methodological and physiological covariates that were shown to covary with hormone levels in the ABCD Study data (Barendse et al., 2024, Herting et al., 2021): time of collection in hours since midnight, the duration of sample collection in minutes, the time from sample collection to placement in freezer storage in minutes, physical activity in the past 12 h (1 =yes; 0 =no), and caffeine intake in the past 12 h (1 =yes; 0 =no).

#### 2.3.5 Sensitivity covariates

Several covariates were included as adjustments in sensitivity analyses (see Analytic Approach below), including age and factors that have been established in the literature to be associated with early pubertal development: body mass index, race/ethnicity, and socioeconomic status. Details on these measures are included in the Supplement. Our goal with these sensitivity analyses was twofold. First, we aimed to evaluate the robustness of the puberty-entropy association to the inclusion of age, as age and pubertal associations are often confounded in the context of brain development (Blakemore et al., 2010; Goddings et al., 2019; Wierenga et al., 2018). Second, because pubertal development (e.g., estimated Tanner stages of 3–5) at the ages examined here (8.92–11.08) is likely the result of *early* pubertal development, we expected the strength of puberty-entropy associations would likely decrease considerably with the inclusion of these covariates (specifically, high adiposity/BMI, low socioeconomic status, and Black/Hispanic racial/ethnic identity) due to over-adjusting the variance of interest (i.e., partitioning out variance in early pubertal development).

### 2.4 Analytic approach

We performed mass univariate linear mixed-effects modeling across all grayordinates (i.e., modeling each cortical vertex and subcortical voxel in turn) using the lmer function from the R package lme4 (Bates et al., 2015) with random effects of family and study site. Family-wise error (FWE) corrected significance thresholds were established by constructing an empirical null distribution of the maximum statistic from 10,000 permutations. Crucially, this permutation strategy respected the nested structure of the ABCD data (family nested within site) by constraining shuffles to appropriate exchangeability blocks. All models were stratified by sex to account for known differences in the timing of the pubertal transition, with analyses of estradiol restricted to female participants. Although subcortical voxels were included in these analyses, our previous work indicates that entropy estimates in these regions are less reliable than in the cortex (Roediger et al., 2024). Consequently, subcortical results are reported in the Supplement, but we broadly did not interpret these results due to robustness concerns.

Analyses were conducted in three phases. First, the mass-univariate models were run regressing entropy values across the whole brain on the pubertal predictor of interest (i.e., approximated Tanner stage, hormone). For all hormone analyses, established confounds (e.g., hours since waking, caffeine) were included across all phases of modeling. In phases two and three, we conducted two sets of sensitivity analyses, as outlined above. Second, these models were refit with the addition of an adjustment for chronological age, to distinguish pubertal effects from age-based effects. Although the ABCD Study sample was designed to constrain variability due to age, findings by Herting and colleagues (2021) indicate that even within the restricted age range of the ABCD baseline timepoint, significant associations between chronological age and pubertal development as operationalized by both pubertal status and hormone levels are evident, suggesting the importance of adjusting for age in evaluating the effects of pubertal development. Finally, the age-adjusted models were further expanded to include several covariates with established connection to early pubertal development (race/ethnicity, family income, parental education, BMI) in order to evaluate the robustness of the findings. As noted above, we expected the inclusion of these covariates to considerably reduce the strength of any identified associations between pubertal development and entropy, as these covariates included are likely over-adjusting for the variance of interest (early pubertal development).

Considering the novel nature of these analyses, we adopted a mixed exploratory-confirmatory approach. We first inspected associations across the cortex, making note of effect sizes. We also applied thresholding to indicate regions with significant associations at α < .05 after FWE correction. To facilitate comparison across pubertal indices (i.e., status, hormones), sexes, and sets of analyses (non-adjusted vs. adjusted), we present standardized beta coefficients in the visualizations of our findings. Consistent with recent recommendations regarding effect sizes in the ABCD Study attempting to replicate “real world” findings (mixed effects analyses, adjustment for covariates), we considered effects of *r* = .03 to be small, *r* = .05 to be medium, and *r* = .09 to be large effects (Owens et al., 2021). In presenting our results, we draw broad comparisons of entropy associations across sexes, pubertal indices, and sets of analyses, leveraging visual inspection of effect size patterns and regions that exhibit significant associations after FWER correction. Finally, in order to better quantify the relative contributions of the indices of pubertal development compared to age and other potentially relevant variables (age; BMI, family income, parental education, race/ethnicity) included in the two sets of sensitivity analyses, for models with substantive associations between pubertal development and entropy we conducted a series of hierarchical leave-out analyses to generate effect sizes at the predictor level. We considered these analyses exploratory and ran them on whole brain entropy (entropy averaged across grayordinates) rather than at the grayordinate level for ease of interpretability.

## 3 Results

**Fig. 2:**
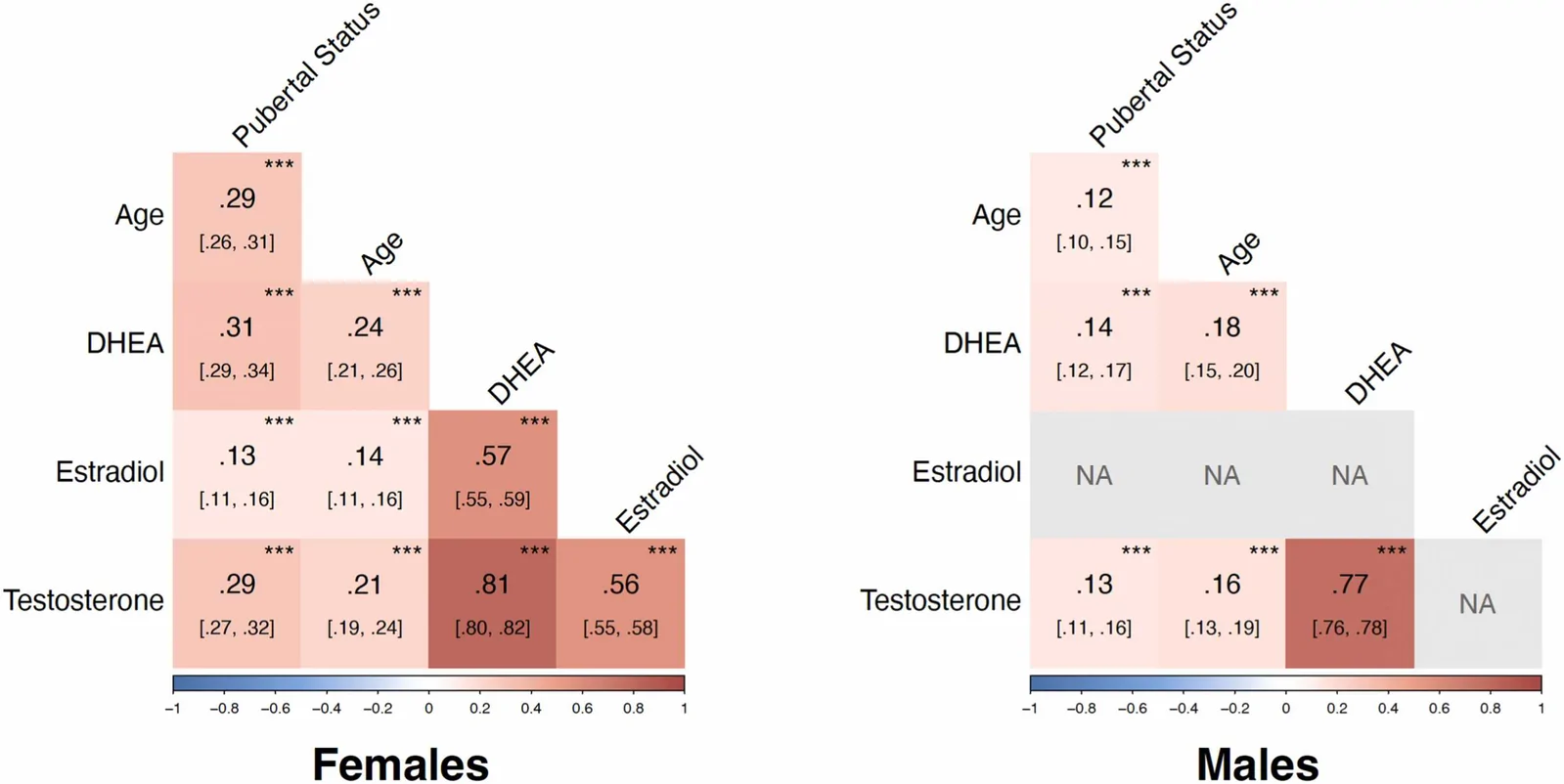
Spearman Correlations of Age and Pubertal Variables, with Confidence Intervals and Split by Biological Sex (*N*_female_ =3444; *N*_male_ = 3394), *Note.* Values in square brackets indicate the 95% confidence interval for each correlation. *** indicates *p* < .001.

**Fig. 3:**
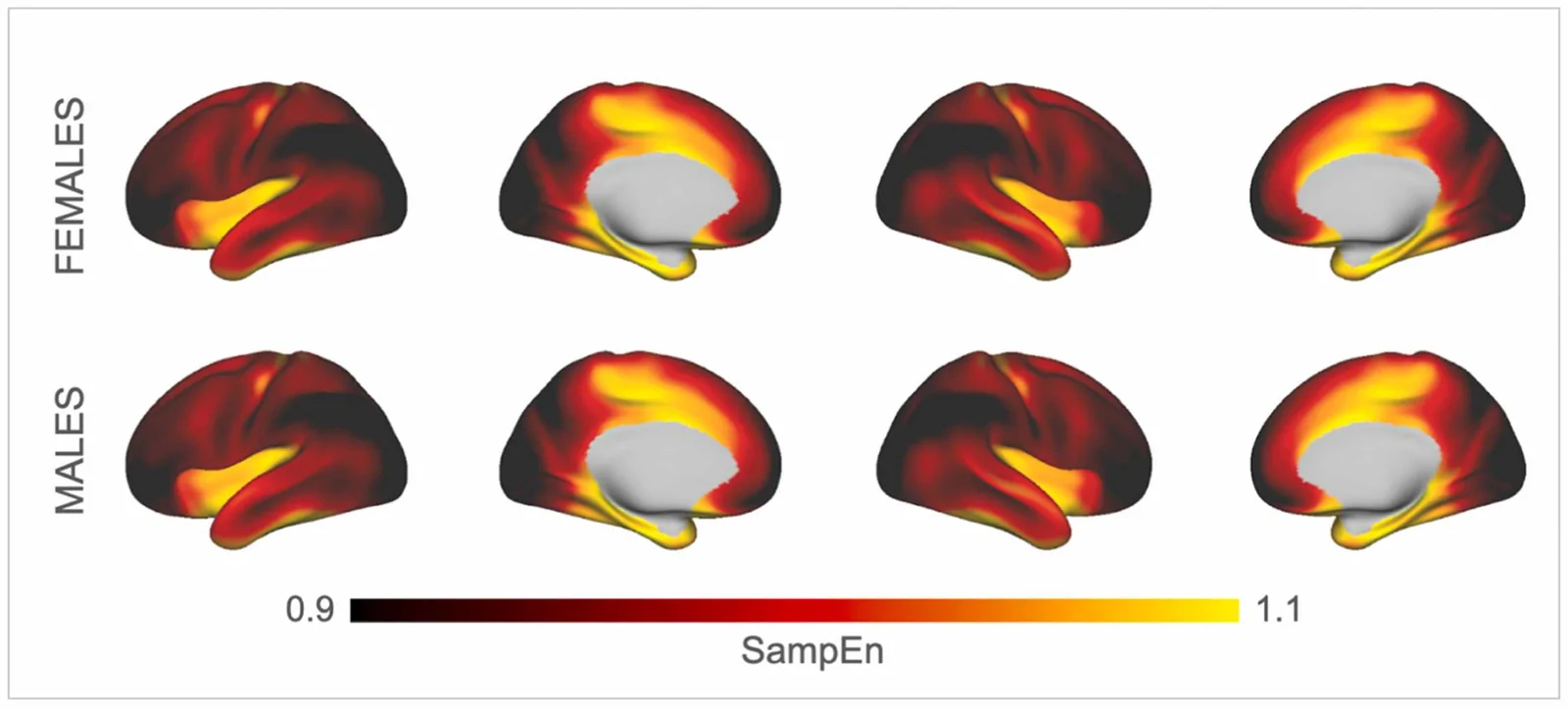
Mean Entropy Values by Biological Sex, *Note*. Higher values indicate greater entropy.

Sample characteristics and descriptive statistics are included in Table 1. As expected, the range of pubertal status was constrained for males compared to females, consistent with typical onset of puberty in males being outside of the age range of the baseline timepoint. Of the entropy subsample, most males were in the pre-pubertal (71%) or early pubertal (23%) stage, with only a handful of males (6%) in the mid- or late-pubertal stages (none were at the postpubertal stage). By comparison, 44% of females were in the mid-, late-, or postpubertal stage. Correlations between primary study variables are presented in Fig. 2; correlations with sensitivity variables are in Figure S1. Age and pubertal status were positively correlated at a large effect size for both females and males. For both sexes, pubertal status was positively correlated with DHEA, testosterone, and for females, estradiol, at large effect sizes. Pubertal hormones were correlated at very large effect sizes for both males and females. Of note, although we have interpreted these effect sizes according to recommendations made in the context of the ABCD Study (see above), they range from small to large on the more traditional approach to interpreting correlations as effect sizes (Cohen, 2013). Fig. 3 depicts mean entropy values by biological sex. Visual inspection indicated similar values between males and females.

**Table 1:** Descriptive Statistics by Sample.

**Full Sample**				**Entropy Subsample**					
**Variable**	***N*****(%)**	***M*****(*****SD*****)**	**min**	**max**	***N*****(%)**	***M*****(*****SD*****)**	**min**	**max**	***SMD (SE)***
Age	11,288	9.91 (0.62)	8.92	11.08	6837	9.96 (0.63)	8.92	11.08	-0.17 (0.02)
Parent Education	11,275	17.14 (2.62)	3.00	21.00	6832	17.33 (2.46)	3.00	21.00	-0.19 (0.02)
Male	5867 (52)				3394 (50)				
Race									
American Indian / Alaska Native	395 (4)				226 (3)				
Asian American	712 (6)				419 (6)				
Black	2299 (20)				1267 (19)				
Native Hawaiian / Pacific Islander	72 (1)				43 (1)				
White	8487 (75)				5324 (78)				
Other	746 (7)				395 (6)				
Entropy	6838 (61)				--				
Puberty Variables									
Pubertal Status	11,289	1.76 (0.87)	1.00	5.00	6838	1.75 (0.86)	1.00	5.00	0.02 (0.02)
Male	5867	1.37 (0.61)	1.00	5.00	3394	1.35 (0.59)	1.00	4.00	-0.07 (0.03)
Female	5422	2.18 (0.91)	1.00	5.00	3444	2.15 (0.90)	1.00	5.00	0.09 (0.03)
DHEA	11,028	62.84 (49.54)	0.00	648.62	6697	63.91 (49.53)	0.00	590.56	-0.06 (0.02)
Male	5710	53.80 (41.64)	0.00	426.63	3327	55.01 (42.57)	0.00	347.68	0.07 (0.03)
Female	5318	72.55 (55.19)	0.00	648.62	3370	72.71 (54.14)	0.00	590.56	-0.01 (0.03)
Testosterone	10,963	33.71 (19.15)	0.00	548.06	6640	33.97 (18.81)	0.00	453.08	-0.03 (0.02)
Male	5690	31.75 (19.86)	0.00	548.06	3300	32.03 (19.31)	0.00	453.08	-0.03 (0.03)
Female	5273	35.82 (18.12)	1.19	205.26	3340	35.89 (18.11)	1.19	205.26	-0.01 (0.03)
Estradiol (female)	5262	1.01 (0.52)	0.00	6.42	3337	1.03 (0.52)	0.00	6.42	-0.11 (0.03)

*Note.* DHEA = dehydroepiandrosterone; *SMD =* standardized mean difference between the full and entropy samples. Participants could endorse multiple racial categories; as a result, counts and percentages total to exceed *N* = 6638/100%.

### 3.1 Entropy associations with approximated Tanner stage

**Fig. 4:**
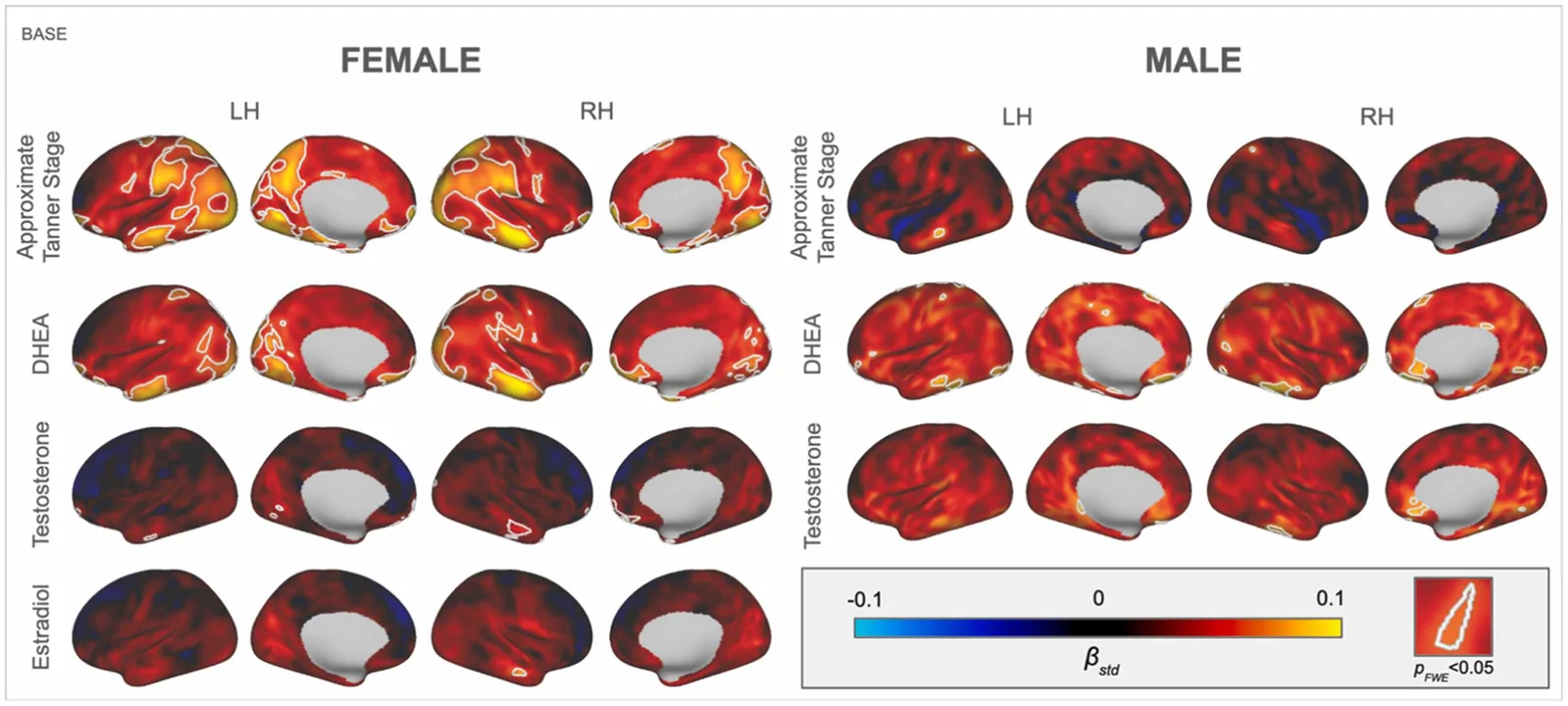
Standardized Associations Between Pubertal Development and Entropy by Biological Sex, *Note*. Areas outlined in white are significant at *p* < .05 after family-wise error (FWE) correction. Estradiol was not collected for male participants. *DHEA* = dehydroepiandrosterone. Hormone analyses were adjusted for time of collection (hours since midnight), the duration of sample collection (minutes), the time from sample collection to placement in freezer storage (minutes), physical activity in the past 12 h, and caffeine intake in the past 12 h.

Positive associations at a medium to large effect size (i.e., approximately *β =*.06 - 0.1) were observed between entropy and pubertal development (as measured by approximated Tanner Stage) over substantial areas of the temporal, parietal, and occipital lobes of the cortex (Fig. 4), as well as in the limbic system and cerebellum (Supplement Figure S4). Although the pattern and direction of association was broadly the same in females and males, correlations were much stronger in females, and only small portions of the temporal and parietal lobes were significant for males after FWER correction. Second, sensitivity analyses adjusting for age showed evidence that associations between approximated Tanner Stage and entropy were still significant, but the significant regions accounted for a smaller area of the cortex for both males and females (Figure S2). Third, as expected, inclusion of additional covariates known to be associated with pubertal development further reduced the magnitude of puberty-entropy associations, with only a few regions remaining significant, and only for females (Figure S3). We conducted an additional exploratory analysis on whole-brain entropy in attempt to shed light on the relative contributions of the additional covariates and pubertal status. BMI accounted for the most variance, followed by race/ethnicity, age, parental education, and family income. Pubertal status did not account for a significant proportion of the variance in whole-brain entropy above and beyond the other covariates for either females or males (see supplement for more detail).

### 3.2 Entropy associations with pubertal hormones

#### 3.2.1 DHEA

For females, the pattern of associations between entropy and DHEA mirrored associations with pubertal status, with significant positive associations in the temporal, parietal, and occipital lobes, as well as the frontal pole, although associations were somewhat weaker for DHEA compared to pubertal status, and smaller portions of the cortex were significant after FWER correction (Fig. 4). For males, by contrast, associations with entropy were somewhat *stronger* for DHEA as compared to associations with pubertal status, particularly in portions of the temporal and occipital lobes, sensorimotor cortex, and frontal pole, with portions of these areas exhibiting significant associations after FWER correction. Second, similar to associations with pubertal status, areas exhibiting significant association were somewhat diminished when adjusting for age (Figure S2). Third, as expected, only very small areas remained significant when adjusting for additional covariates (Figure S3). Given the significant associations between DHEA and entropy observed in both females and males, we conducted an exploratory analysis on whole-brain entropy to examine the relative contributions of DHEA and sociodemographic covariates. For females, similar to the models for pubertal status, BMI explained the most variance in entropy, followed by race/ethnicity, age, parental education, and income, followed by DHEA, which did not explain a significant portion of the variance. For males, findings differed somewhat. BMI again explained the largest proportion of variance in entropy, followed by race/ethnicity and parental education. However, DHEA explained a significant proportion of variance in whole-brain entropy, smaller than that of the aforementioned covariates, but larger than that for parental income and age (see supplement for detail).

#### 3.2.2 Testosterone

For females, associations between entropy and testosterone were weaker than associations observed between entropy and both pubertal stage and DHEA (Fig. 4), with significant associations in small areas of the anterior temporal lobe, the occipital lobe, and a small portion of the frontal pole. For males, entropy-testosterone associations were similar to DHEA: Somewhat stronger than entropy-pubertal status associations across the brain, with small regions observed to exhibit significant associations after FWER correction, specifically in the right anterior temporal lobe, the right ventromedial frontal lobe, and the frontal pole. Again, paralleling the pattern of associations from sensitivity analyses with pubertal status and DHEA, adjustment for age reduced the magnitude of associations (Figure S2). For females, almost no associations survived age adjustment and FWER correction. For males, areas of significance remained but were reduced in size when adjusting for age. No entropy-testosterone associations remained significant when adjusting for sociodemographic covariates (Figure S3).

#### 3.2.3 Estradiol (Females only)

For females, associations between entropy and estradiol were yet weaker than associations between entropy and pubertal stage, DHEA, and testosterone, with the strongest association evident in the right anterior temporal pole (Fig. 4). The size of the region of significant association was reduced in the sensitivity analysis adjusting for age (Figure S2), and no associations remained significant in the sensitivity analysis adjusting for sociodemographic covariates (Figure S3).

## 4 Discussion

In this novel set of analyses, we report that entropy of the fMRI BOLD signal during resting-state increases with early pubertal development across a range of cortical brain areas that are implicated in adolescent brain development. As a metric of complexity, the entropy of the brain’s fMRI signals during rest has been increasingly studied throughout the lifespan as an indicator of brain development (Cieri et al., 2021). Building on theories of brain development that center puberty as a critical process in spurring reorganization in brain structure and function throughout adolescence, and especially recent work raising the potential importance of adrenarche, this study examined for the first time how brain entropy is associated with early pubertal development. Key study strengths include the large, diverse sample of children in a narrow developmental age range of interest for this research question, and a large amount of resting-state fMRI data allowing for advanced analyses to measure brain entropy with rigor. Results indicated that higher levels of entropy were associated with more advanced pubertal development in this sample of youth ages 8.92–11.08 years, with the strongest associations observed when puberty was measured by pubertal status (as reported by parents) and DHEA salivary concentrations. The pattern of associations aligns with established research on functional brain development in adolescence (Crone and Dahl, 2012, Nelson et al., 2016), supporting the notion that entropy may be one marker of the opening of various neural systems at puberty (Crone and Dahl, 2012, Larsen and Luna, 2018). The planned sensitivity analyses suggest that entropy-puberty associations are complex at this developmental stage, and our findings do not preclude the possibility that other factors (e.g., age, BMI, SES) evaluated here may play a direct or independent role. Hence, we consider these interpretations tentative. Longitudinal research will be necessary to disentangle whether higher levels of entropy are, indeed, associated with of greater pubertal development, or if some combination of the additional factors evaluated here in sensitivity analyses (e.g., age, SES, BMI) better account for the changes.

Notably, there was evidence for puberty-entropy associations across various indices of pubertal development (i.e., status, hormones). Puberty-entropy associations were considerably stronger in females compared to males, likely reflecting – at least in part – normative sex differences in pubertal timing. Females start puberty approximately two years before males (Berenbaum et al., 2015), and 44% of females in this sample reported they were in the “midpubertal” stage or later. This interpretation is broadly supported by the pattern of findings *across* pubertal indicators: For females, pubertal associations with entropy were strongest with parent-reported pubertal status, which likely captures aggregate development (i.e., beyond single-hormone concentrations), although some associations were observed for hormones as well, and particularly with DHEA. For most of the males in this sample, the outward signs of puberty were largely not evident (only 29% of males were reported to have begun puberty at all). Instead, the strongest associations were observed with DHEA – the earliest pubertal hormone to exhibit substantive increases, and which may increase in males even prior to visible pubertal development (Byrne et al., 2017). Weaker associations were observed with testosterone and estradiol (in females); these hormones typically exhibit increases later in puberty (Berenbaum et al., 2015).

Several neurobiological changes associated with early pubertal development may account for the associations observed here. First, higher levels of entropy may be related to increases in adolescent neuroplasticity beginning at puberty, which have been shown to relate to alterations in pubertal hormones across animal and human studies (Larsen and Luna, 2018, Parr et al., 2024). Additionally, considerable development occurs along the sensorimotor-association axis during puberty (Gogtay et al., 2004, Larsen et al., 2023, Toga et al., 2006), paralleling findings on neural entropy and earlier stages of sensorimotor-association axis development in infancy (Zhao et al., 2024). Indeed, findings observed here appear to broadly map on to regions along the sensorimotor-association axis that develop in early puberty, including regions of the parietal and temporal cortices (Behrmann et al., 2004, Larsen et al., 2023, Northoff et al., 2006, Van Overwalle, 2009), which play a role in sensory integration, attention, self-referential processing, and social cognition. These regions of the association cortex are the last to fully myelinate and show long-range functional connectivity and high levels of between-person variability in functional connectivity (Buckner and Krienen, 2013). Thus, it may be that neural entropy facilitates or is a marker of increased plasticity in regions maturing during this stage of late childhood and early adolescence. If this is the case, we would expect entropy to continue increasing in the frontal lobe as pubertal development advances.

Entropy-puberty associations were strongest when puberty was operationalized as estimated Tanner stage (for females) and DHEA salivary concentration (for males and females). Intriguingly, entropy associations for males, specifically, were more strongly associated with DHEA than estimated Tanner stage, and DHEA-entropy associations for females largely mirrored Tanner stage-entropy associations, albeit at somewhat smaller effect sizes. These findings may indicate a role of DHEA in entropy differences during pubertal development. Most research on hormone influences on brain development has focused on the influence of largely sex-specific gonadal hormones (i.e., estradiol, testosterone; Schulz and Sisk, 2016). However, recent research raises the possibility that adrenarche, and the rise in DHEA that it brings, may play an important role in brain development (Barendse et al., 2018, Byrne et al., 2017), potentially through neural entropy. Adrenarche is associated with increases in peripherally circulating and brain-synthesized DHEA and its sulfate conjugate (DHEA-S). Adrenarche has historically been predominantly associated with increases in body odor and growth in height; however, more recently scholars have proposed that adrenarche and DHEA may be associated with increased brain plasticity, not only during puberty but also in early development and later life (Byrne et al., 2017, Cumberland et al., 2021). Of note, most murine and rodent models, on which the most prominent theories of the role of sex hormones in brain development are based (e.g., Schulz et al., 2009), do not experience a prolonged adrenarche; the process is almost exclusively observed in higher order primates (Byrne et al., 2017, Conley et al., 2012). Hence, these results may point to the importance of adrenarche in preparing the brain for the development/reorganization to come. In other words, we may speculate that DHEA is “laying the stage” for context-dependent learning and the reorganizational/activational effects of sex-specific hormones, with entropy as a mediating biological marker. Along these lines, the relatively stronger DHEA-entropy associations observed in females versus males may reflect more protracted influence of DHEA in the brain (i.e., the effects of heightened circulating/brain-derived DHEA aggregating over time; Byrne et al., 2017). Hence, an important next step will be tracking these changes longitudinally in later waves of the ABCD study, with special attention to the potential confounds of age, SES, and BMI, as noted.

Although we conceptualize that pubertal associations with entropy are best explained by biological changes driven by pubertal development, rather than direct effects of age, socioeconomic status, or BMI on entropy, this study has not been designed to fully rule out that possibility. The planned sensitivity analyses to some degree bolster the interpretation that pubertal development is associated with entropy; however, the cross-sectional nature of these analyses raises the limitation of potential confounding variables. These sensitivity analyses reflect a tentative underlying causal model in which puberty may play a mechanistic role driving increases in entropy, with the caveat that the *early pubertal development* we observe in this sample at these ages is likely causally driven, in turn, by demographic (SES, race/ethnicity) and body composition factors (BMI), as has been robustly established in the pubertal development literature (e.g., Carter et al., 2013; Feng and Ren, 2025). Although we believe the preponderance of the evidence presented here supports early emerging associations between pubertal development and entropy, the observed differences across males and females and reduced predictive power of pubertal measures exhibited in sensitivity analyses raise alternative explanations. It may be that the entropy associations are instead attributable to experiences related to age, SES, and/or physiological changes associated with elevated BMI in late childhood and prevalent primarily in females. BMI, specifically, warrants more attention in future work, given it accounted for the most variance explained in whole-brain entropy in sensitivity analyses. BMI was highly correlated with pubertal status and DHEA in this sample, and it may be a stronger indicator or predictor of early puberty than age or any of the other sociodemograhpic variables associated with early puberty. There is increasing recognition of the role of high BMI in early pubertal development (Feng and Ren, 2025), and these findings may suggest that BMI functions as a leading indicator of pubertal change (e.g., weight gain before growth spurts, increasing BMI as a marker of development).

Although associations were less robust to adjustment for covariates known to be associated with early pubertal development, associations were broadly robust to the inclusion of chronological age. Disentangling pubertal associations from age- or experience-related associations is a perennial challenge of adolescent (neuro)development research; yet the implication is that associations with entropy observed here – at least for females, where associations were more robust – are broadly attributable to puberty rather than age (although age did appear to account for some of the variance in entropy, perhaps unsurprisingly). On that note, it is possible, and perhaps likely, that there may be *both* age- and puberty-based increases in entropy in considering empirical findings in infants (Zhao et al., 2024) and children (Amalric and Cantlon, 2023), that suggest that entropy increases with age and functional improvement on cognitive tasks. Hence, future longitudinal work examining these associations will be most impactful if it is carefully designed with regard to age *and* pubertal development (and likely their interaction).

Now that preliminary entropy-pubertal development associations have been established, longitudinal studies are required to further elucidate how entropy changes across the full span of pubertal development, both to delineate developmental trajectories as well as to disentangle the potential contributions of sociodemographic variables, including those examined here, from associations directly attributable to pubertal development. Ideally, such studies will not only examine cross-sectional associations at various ages/pubertal stages, but will examine within-person longitudinal change as well, considering several associations between pubertal and brain development first identified cross-sectionally differed when examined longitudinally (Vijayakumar et al., 2018).

### 4.1 Limitations & future directions

There are several limitations to note regarding the current findings. As noted above, in contrast with models adjusting for age, we found that puberty-entropy associations did not generally survive adjustment for variables associated with early pubertal development. At the ages of the baseline ABCD assessment (here, ages 8.92–11.08), much of the variability in pubertal development represents *early* pubertal development (i.e., early in age compared to one's peers) and early pubertal development is highly correlated with high BMI, race/ethnicity, and socioeconomic status (see correlation table in the supplemental material). Thus, we anticipated that much of the variance in early pubertal development observed would be attributable to participants’ sociodemographic experiences and physical characteristics. Though early pubertal development may be interconnected with these factors (i.e., socioeconomic status, BMI, race/ethnicity), we believe that the associations with entropy are largely explained by the biological alterations driven by pubertal development, rather than some other mechanism directly linking these experiences (i.e., socioeconomic status, BMI) to brain development. We anticipate that as more normative (i.e., on time/late vs. early) pubertal development becomes evident at later waves of the ABCD data, these factors will have less of an impact on the puberty-entropy associations. However, we acknowledge that is an empirical question that requires evaluation in future work. Indeed, it may be that these experiences, such as having low SES or high BMI, may have direct impacts on brain development at these ages through alternative pathways. In support of such direct associations, other studies examining socioeconomic status associations with functional connectivity in ABCD (e.g., Marek et al., 2026; Rakesh et al., 2021) and other samples (Tooley et al., 2021) have found large associations between socioeconomic status and connectivity. However, such studies have typically found similar effect sizes in males and females. Here, we interpret the considerably smaller effects in males (and strongest associations with DHEA, as explicated above) as consistent with the comparative later start to puberty in males and potentially due to less variability in pubertal measures; nonetheless, we once again note that there may be direct links between socioeconomic status or BMI and entropy, rather than associations primarily mediated through pubertal development, which will be necessary to establish through longitudinal work.

In addition to these confounds, the sample had limited variability in pubertal development in males, and somewhat constrained pubertal development in females. While these results provide initial support for puberty-entropy associations, the lack of variability in male pubertal development at the ages captured at baseline (i.e., 8.92–11.08. years) necessitates further investigation to determine whether the findings here reflect either a) time-lagged differences in development due to sex differences in pubertal timing, or b) true sex differences in puberty-entropy associations. We hypothesize that associations between entropy and pubertal development will strengthen and become more evident within ABCD’s longitudinal data as more male participants will have experienced pubertal changes. However, this is a tentative hypothesis based on known differences in the timing of pubertal development; it is also possible that entropy may unfold in a different manner for males versus females, particularly as there is increased circulation of sex-specific hormones in later adolescence (Schulz and Sisk, 2016, Vijayakumar et al., 2018).

Second, although strengths of this study include the large, diverse sample; standardized fMRI protocol; and the simultaneous examination of multiple measures of pubertal development (status, hormones), there are limitations to each of the pubertal measures used. As noted above, the pubertal status measures used here was derived from parental reports and the hormone measures reflect a singular snapshot of hormone levels in the context of known fluctuations, particularly diurnal and contextual variations in testosterone and menstrual variation in estradiol. Hence, it is likely we have missed important within-person variation in these processes, and our (between-person) estimates likely contain more noise than may be ideal, due to systematic within-person variation. (See Beltz and colleagues, [Beltz et al., 2026], for a critical perspective on the use of pubertal measures in the ABCD Study, and particularly salivary hormones.) Despite these measurement limitations, our findings establish foundational interrelations between pubertal development and entropy in a large, diverse sample, findings that should spur future study – ideally with rigorous, within-person operationalizations of pubertal development – to further characterize these associations.

Finally, entropy estimates in this sample in subcortical areas are less reliable than estimates from cortical areas (Roediger et al., 2024); as such, we have not interpreted those findings. However, considerable reorganization happens in limbic structures and corticolimbic networks during adolescence. Research identifying ways to increase the reliability of entropy estimates in subcortical structures is an important next step, as is replication of these findings in other samples as well as longitudinally within the ABCD sample to evaluate their robustness.

### 4.2 Conclusion

This study provides the first evidence, to our knowledge, of changes in brain entropy associated with early pubertal development. We hope that these findings will spur rigorous theory- and hypothesis-driven research designed to chart the developmental unfolding of changes in brain entropy. Ideally, such studies will have sensitivity to the overlapping and interactive pubertal processes that may play a role in entropy changes, spanning both specific hormone trajectories as well as broader measures such as pubertal status. Such studies will be most informative if they are designed in a fashion that is sensitive to sex, pubertal stage, *and* age, and informed by the literature with regard to established associations between pubertal development, age, and change in functional ability.

## CRediT authorship contribution statement

**McKone Kirsten M.P.:** Writing – review & editing, Writing – original draft, Methodology, Formal analysis, Conceptualization. **Donovan J. Roediger:** Writing – review & editing, Writing – original draft, Visualization, Software, Methodology, Investigation, Formal analysis, Data curation, Conceptualization. **Stefanie L. Sequeira:** Writing – review & editing, Writing – original draft, Methodology, Conceptualization. **Ellery Island:** Writing – review & editing, Methodology, Data curation. **Monica Luciana:** Writing – review & editing, Writing – original draft, Methodology, Funding acquisition, Conceptualization. **Mark B. Fiecas:** Writing – review & editing, Writing – original draft, Methodology, Funding acquisition, Data curation, Conceptualization. **Bryon A. Mueller:** Writing – review & editing, Methodology, Funding acquisition, Conceptualization. **Bonnie Klimes-Dougan:** Writing – review & editing, Writing – original draft, Supervision, Methodology, Funding acquisition, Conceptualization. **Kathryn R. Cullen:** Writing – review & editing, Writing – original draft, Supervision, Methodology, Funding acquisition, Conceptualization.

## Data Statement

Data used in the preparation of this article were obtained from the Adolescent Brain Cognitive Development (ABCD) Study (https://abcdstudy.org), held in the NIMH Data Archive (NDA). This is a multisite, longitudinal study designed to recruit > 10,000 children age 9–10 and follow them over 10 years into early adulthood. The ABCD Study® is supported by the 10.13039/100000002National Institutes of Health and additional federal partners under award numbers U01DA041048, U01DA050989, U01DA051016, U01DA041022, U01DA051018, U01DA051037, U01DA050987, U01DA041174, U01DA041106, U01DA041117, U01DA041028, U01DA041134, U01DA050988, U01DA051039, U01DA041156, U01DA041025, U01DA041120, U01DA051038, U01DA041148, U01DA041093, U01DA041089, U24DA041123, U24DA041147. A full list of supporters is available at https://abcdstudy.org/federal-partners.html. A listing of participating sites and a complete listing of the study investigators can be found at https://abcdstudy.org/consortium_members/. ABCD consortium investigators designed and implemented the study and/or provided data but did not necessarily participate in the analysis or writing of this report. This manuscript reflects the views of the authors and may not reflect the opinions or views of the NIH or ABCD consortium investigators. The ABCD data repository grows and changes over time. The demographic ABCD data used in this report came from Data Release Version 5.1. For the imaging data, the raw DICOM data were downloaded from the ABCD FastTrack release and then processed using version 0.1.3 of the ABCD-BIDS pipeline from the Developmental Cognition and Neuroimaging (DCAN) Lab at the University of Minnesota. Further information on this pipeline can be found at https://github.com/DCAN-Labs/abcd-hcp-pipeline.

## Declaration of Competing Interest

All authors declare no competing or conflicting interests.

## Data Availability

Data are publicly available at https://nda.nih.gov/abcd.
