# Supplementary material for: Same ages, different stages: Pubertal development in 9- and 10-year olds is associated with entropy of fMRI signals
Source: Dev Cogn Neurosci. 2026 Sep 1;81:101806. doi: 10.1016/j.dcn.2026.101806 (PMC13571737; doi:10.1016/j.dcn.2026.101806)
Supplement: Supplementary file 1 [file mmc1.docx]

**Supplemental Information**

**Measures**

**Sensitivity Covariates**

**Body Mass Index (BMI).**

Body mass index z-scores (BMIz) were calculated from measurements of height and weight taken at the baseline visit using professional-grade equipment, using the child’s age and sex using the United States’ 2000 Center for Disease Control (CDC) growth charts. There were several participants that appeared to have erroneous data in either height or weight (e.g., implausibly low weight, height of 6’10”) that resulted in very low BMIz (e.g., BMIz < -5SD). As a result, outliers were changed to missing if the participant had a height above or below the first percentile *or* weight below the first percentile *and* a BMIz below the first percentile.

**Race and Ethnicity.**

Participants identified their racial and ethnic identification through a multi-select series of check boxes. Dummy codes (1=endorsement of the category; 0=no endorsement) were created for the following non-exclusive categories: American Indian or Alaska Native; Asian American; Black or African American; Native Hawaiian or Pacific Islander; other racial identification; White. “Other” racial identification was used as the reference group. Hispanic identity was similarly coded (1=endorsement of the category; 0=no endorsement) and entered as a separate variable.

**Highest Parental Education.**

Participants’ primary caregivers reported their highest level of education attained. Highest parental education was translated into a series of dummy codes indicating categories of (1) less than high school diploma, (2) high school diploma or GED, (3) some college or associates degree, (4) bachelor’s degree, and (5) postgraduate degree, with high school diploma or GED used as the reference group.

**Household Income.**

Participants reported their household income to the nearest $10,000 increment. Dummy coded variables were generated for categories of income of $49,999 or less, $50,000 to $99,999, and $100,000+, with the middle category as the reference group.

**Results**

**Exploratory Sensitivity Analysis on Whole Brain Entropy**

An additional exploratory sensitivity analysis was conducted leveraging whole brain entropy (entropy averaged across grayordinates) with the aim of establishing the relative contributions of pubertal status and DHEA (the pubertal metrics exhibiting the strongest associations with entropy in females and males, respectively). We used hierarchical block subtraction to generate estimates of the effect size in variance explained of each set of predictors.

***Pubertal Status***

Models for pubertal status indicated that BMI accounted for the most variance explained in whole-brain entropy and explained a significant proportion of the variance. The variable block for race/ethnicity accounted for the next largest proportion of the variance in the aggregate, which was significant for males. For females, none of the dummy coded race variables accounted for a significant proportion of the variance. Age accounted for the next largest proportion of variance and was significant, followed by parental education, family income, and pubertal status, none of which predicted significant proportions of the variance.

***DHEA***

Similar to the models for pubertal status, for both males and females, BMI explained the largest significant proportion of variance, followed by race/ethnicity, which explained a significant proportion of the variance for males but not females. However, for the remainder of the predictors, variance explained diverged by sex. For females, age explained the next largest, significant proportion of the variance, followed by parental education, family income, and DHEA, none of which explained significant proportions of the variance. For males, education explained the next largest, but non-significant proportion of the variance, followed by DHEA and age, which each explained a significant proportion of the variance, and family income, which was not significant.

Figure S1. Spearman Correlations of Age, Pubertal Variables, and Sensitivity Variables by Sex (*N*_female_ =3,444 ; *N*_male_ = 3,394)

*Note.* Values in square brackets indicate the 95% confidence interval for each correlation.

** indicates *p* < .01. *** indicates *p* < .001.

Figure S2. Standardized Age-adjusted Associations Between Pubertal Development and Entropy by Sex

*Note*. Areas outlined in white are significant at *p* < .05 after family-wise error (FWE) correction. Estradiol was not collected for male participants. *DHEA* = dehydroepiandrosterone. Hormone analyses were adjusted for time of collection (hours since midnight), the duration of sample collection (minutes), the time from sample collection to placement in freezer storage (minutes), physical activity in the past 12 hours, and caffeine intake in the past 12 hours.

Figure S3. Standardized Demographic- and Age-adjusted Associations Between Pubertal Development and Entropy by Sex*Note*. Areas outlined in white are significant at *p* < .05 after family-wise error (FWE) correction. Demographic variables include race, ethnicity, body mass index (z-scored), household income, and parental education. Estradiol was not collected for male participants. *DHEA* = dehydroepiandrosterone. Hormone analyses were adjusted for time of collection (hours since midnight), the duration of sample collection (minutes), the time from sample collection to placement in freezer storage (minutes), physical activity in the past 12 hours, and caffeine intake in the past 12 hours.

**Figures S4-S6. Standardized Associations Between Entropy and Pubertal Development in Subcortical Regions.**

Figure S4. Standardized Subcortical Puberty-Entropy Associations by Sex

*Note*. Areas outlined in white are significant at *p* < .05 after family-wise error (FWE) correction. Estradiol was not collected for male participants. *DHEA* = dehydroepiandrosterone. Hormone analyses were adjusted for time of collection (hours since midnight), the duration of sample collection (minutes), the time from sample collection to placement in freezer storage (minutes), physical activity in the past 12 hours, and caffeine intake in the past 12 hours.

Figure S5. Standardized Subcortical Puberty-Entropy Associations by Sex with Adjustment for Age

*Note*. Areas outlined in white are significant at *p* < .05 after family-wise error (FWE) correction. Estradiol was not collected for male participants. *DHEA* = dehydroepiandrosterone. Hormone analyses were adjusted for time of collection (hours since midnight), the duration of sample collection (minutes), the time from sample collection to placement in freezer storage (minutes), physical activity in the past 12 hours, and caffeine intake in the past 12 hours.

Figure S6. Standardized Subcortical Puberty-Entropy Associations by Sex with Adjustment for Age and Demographic Covariates

*Note*. Areas outlined in white are significant at *p* < .05 after family-wise error (FWE) correction. Demographic variables include race, ethnicity, body mass index (z-scored), household income, and parental education. Estradiol was not collected for male participants. *DHEA* = dehydroepiandrosterone. Hormone analyses were adjusted for time of collection (hours since midnight), the duration of sample collection (minutes), the time from sample collection to placement in freezer storage (minutes), physical activity in the past 12 hours, and caffeine intake in the past 12 hours.
